# Effect of propolis supplementation on athletic performance, body composition, inflammation, and oxidative stress following intense exercise: A triple‐blind randomized clinical trial

**DOI:** 10.1002/fsn3.2319

**Published:** 2021-05-08

**Authors:** Davood Soleimani, Mahsa Miryan, Vahid Hadi, Jamshid Gholizadeh Navashenaq, Jalal Moludi, Sayed Mazaher Sayedi, Mohammad Bagherniya, Gholamreza Askari, Seyyed Mostafa Nachvak, Ehsan Sadeghi, Ali Ashraf Rashidi, Saeid Hadi

**Affiliations:** ^1^ Department of Health, Science and Research Branch AJA University of Medical Sciences Tehran Iran; ^2^ Nutritional Sciences Department, School of Nutrition Sciences and Food Technology Kermanshah University of Medical Sciences Kermanshah Iran; ^3^ Department of Clinical Nutrition, School of Nutrition and Food Sciences Tabriz University of Medical Sciences Tabriz Iran; ^4^ Noncommunicable Diseases Research Center Bam University of Medical Sciences Bam Iran; ^5^ Department of Community Nutrition, School of Nutrition and Food Science Isfahan University of Medical Sciences Isfahan Iran; ^6^ Research Center for Environmental Determinants of Health (RCEDH) Kermanshah University of medical Sciences Kermanshah Iran

**Keywords:** athletic performance, body composition, inflammation, oxidative stress, propolis, VO_2_ max

## Abstract

**Background:**

Emerging evidence indicates that propolis as a novel potential antioxidant has unique benefits. This study aimed to evaluate the effect of propolis on oxidative stress, inflammation, body composition, and athletic performance in healthy active subjects.

**Methods:**

This clinical trial was conducted on 54 male military cadets. Eligible subjects were randomly allocated to receive a single dose of 450 mg propolis twice daily for four weeks or a matching placebo containing microcrystalline cellulose. Cooper 12‐min run test and running‐based anaerobic sprint test were performed to measure aerobic and anaerobic performance. Blood samples were obtained immediately after Cooper's test to evaluate oxidative stress and inflammation status. Fat mass and fat‐free mass were analyzed using bioelectrical impedance.

**Results:**

Mean changes in fat mass, fat‐free mass, anaerobic powers, fatigue index, and VO_2_ max did not differ significantly between the two groups after the adjustment for baseline values (P‐value>0.05). A significant change was observed in plasma levels of IL‐6 (−1.43 ± 0.11pg/mL), total oxidant status (−3.9 ± 0.2µmol/L), total antioxidant capacity (164 ± 12 µmol/L), malondialdehyde (−0.52 ± 0.03µmol/L), oxidative stress index (−0.45 ± 0.04), and glutathione (48.72±2µmol/L) in the propolis group compared with the placebo group after the adjustment for baseline values and weight changes (P‐value<0.05). Although IL‐10 concentrations had no significant changes in both groups, the ratio of IL‐6/IL‐10 significantly reduced in the propolis group compared with the placebo group (−0.174 ± 0.015 versus. 0.051 ± 0.014; P‐value: 0.041).

**Conclusions:**

Our results indicated that propolis might have beneficial effects on oxidative stress and inflammation following intense activities in healthy male subjects.

## Graphical abstract

As far as we know, this is the first trial to evaluate the effect of propolis supplementation on exercise performance. This clinical trial study shows that propolis supplementation can significantly reduce plasma levels of total oxidants, IL‐6, malondialdehyde, oxidative stress index, and IL‐6/IL‐10 ratio and can significantly improve plasma levels of total antioxidant capacity and glutathione following intense physical activity. However, our trial indicates that propolis intake had no significant effect on anaerobic and aerobic performance and body composition.

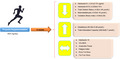

## 1 INTRODUCTION

Intense physical activities can increase oxygen consumption in the active muscles 10–15 times more than in the resting state (Joyner & Case y, 2015). The rise in oxygen delivery to active skeletal muscles is required to produce adenosine triphosphate (ATP) through the electron transport chain (ETC) to continue the activities (Miyazaki et al., 2001). The ETC is located in the inner mitochondrial membrane, which is not only the principal site of ATP production in skeletal muscles but also a potential source of reactive oxygen species (ROS) production (Leeuwenburgh & Heinecke, 2001). In addition to ETC, xanthine oxidase, lipoxygenase, phospholipase A_2_, myostatin, and catecholamines also contribute to ROS formation in contracting muscles (Powers & Jackson, 2008; Steinbacher & Eckl, 2015).

Some researchers have suggested that low ROS concentrations in skeletal muscles can increase the muscle's ability to produce force (Reid, 2001). However, what is certain is that ROS in high concentrations results in muscle force decline and fatigue (Powers et al., 2016). In addition, overproduction of ROS following intense activates can cause irreversible damage to intracellular organelles, inflammation, and eventually muscle breakdown (Suzuki et al., 2020). The susceptibility of skeletal muscle to oxidative stress depends on the antioxidant defense system capability. This system comprises nonenzymatic antioxidants, such as glutathione (GSH), and enzymatic antioxidants such as superoxide dismutase (SOD) and catalase (CAT). Nutritional status influences the capacity and ability of the body's antioxidant defense to neutralize ROS (Chow, 1979). In addition, dietary antioxidants can modulate the immune response and inflammatory pathways which can help the enhancement of physical performance and recovery from exercise in athletes (Mason et al., 2020).

Propolis is a sticky substance produced by honey bees (*Apis mellifera L*.) from buds and various plants' exudates. Numerous polyphenol compounds have been identified in propolis from different geographical regions of the world (Huang et al., 2014). Propolis has long been used as a popular therapeutic agent to promote the body's health and treat wounds and infections (Sforcin, 2016). Recent studies have shown the antioxidant, anti‐inflammatory, and immunomodulatory activities of propolis from different parts of the world (Franchin et al., 2018; Soleimani, Miryan, et al., 2021; Wang et al., 2018). Animal studies support that propolis and its derivatives can ameliorate exercise‐induced damage through enhancing the antioxidant defense system and suppressing nuclear factor‐kappa B (NF‐κB) (Kwon et al., 2014; Shen et al., 2013). Quercetin, the predominant flavonoid compound identified in propolis (Zheng et al., 2017), has been shown to promote exercise performance (Davis et al., 2010) and muscle mitochondrial biogenesis (Islam et al., 2020). This evidence indicates that propolis might help athletes protect their muscles against exercise‐induced oxidative and inflammatory damage and improve exercise performance. The current trial aimed to assess the effect of propolis supplementation on pro/anti‐inflammatory cytokines, antioxidant/oxidant status, and anaerobic/aerobic endurance among active subjects.

## 2 METHODS AND MATERIALS

### 2.1 Study design and participants

**FIGURE 1:**
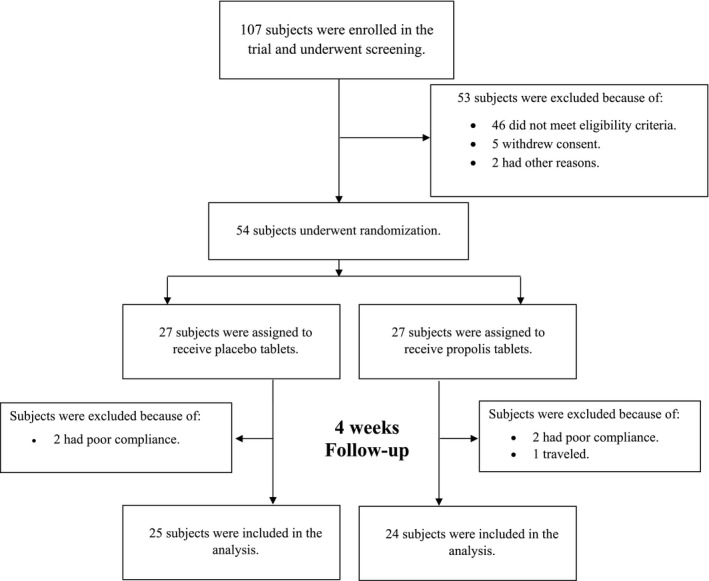
Screening, Randomization, Treatment, and Follow‐up

This randomized, triple‐blind, placebo‐controlled clinical trial was designed to evaluate the effect of propolis supplementation on pro/anti‐inflammatory cytokines, antioxidant/oxidant status, and exercise performance. Participants were recruited from new male cadets entering AJA University of Medical Sciences in Tehran, Iran. A total of 107 young males were enrolled in the trial, of whom 54 eligible participants underwent randomization to assign either the propolis group or the placebo group (Figure 1). The protocol, available at the Iranian Registry of Clinical Trials (IRCT20180824040857N2), was reviewed and approved by the ethics committee at the AJA University of Medical Sciences (IR.AJAUMS.REC.1399.107). Each participant was provided verbally with information on the objectives of the trial and its benefits and possible health risks at the time of enrollment. Each participant provided written informed consent. This trial was conducted in accordance with the principles of the Helsinki Declaration.

### 2.2 Eligibility criteria

Cadets, who agreed to participate in this trial, were screened based on eligibility criteria. Inclusion criteria were male gender, age of 20–30 years, body mass index (BMI) of 18.5–25 kg/m^2^, a minimum of 6 hr/wk of sports activities in the last year, and written informed consent. Those who had a history of adverse reaction to bee products, musculoskeletal dysfunction, previous musculoskeletal injuries during training or exercise, heart diseases, diabetes mellitus, metabolic diseases, regular use of antioxidant supplements (e.g., vitamin E, vitamin C, β‐carotene, selenium, and propolis) or anti‐inflammatory drugs in the last three months, or specific dietary regimen (e.g., vegetarian diets) and energy‐restricted diets, in the last six months were not eligible to participate in the trial.

### 2.3 Trial randomization and blinding

Cadets who met the eligibility criteria underwent randomization after the screening visit. Randomization sequences were generated using a random‐number table and opaque, sealed, numbered envelopes. Randomization was stratified according to age (20–25 years versus. 26–30 years). Eligible participants were randomly assigned in a 1:1 ratio with a blinded manner to the intervention or control group. Participants and investigators, except the trial pharmacist, were concealed from the study‐group assignment until the end of the trial and data analyses.

### 2.4 Intervention

Participants in the intervention group were assigned to take ethanolic‐extracted poplar propolis at a single dose of 450 mg twice daily, before lunch and dinner, for 4 weeks. In contrast, those in the control group received a matching placebo containing microcrystalline cellulose. The dose of propolis was selected according to the previous phase ΙΙ trial (Zhao et al., 2016). Propolis extract was standardized based on total polyphenols and flavonoids content, according to Bankova's recommendation (Bankova, 2005). Each propolis tablet contained 180 mg polyphenols and 134 mg flavonoids. The Reyhan Naghsh Jahan Pharmaceutical Company manufactured both the propolis and matching placebo tablets under good manufacturing practice (GMP) conditions. Adherence was estimated by counting unused tablets. During the trial, all cadets were in the same place, had the same physical activity, ate the same meals, and slept the same number of hours. Participants were also followed by face‐to‐face visits every week. During the intervention period, participants who were unwilling to continue the trial were sensitive or unable to take the assigned intervention (<80%), or used other dietary supplements and drugs were excluded from the trial. Among 54 eligible participants, 49 completed the 4‐week intervention period. Two subjects in the propolis group and two subjects in the placebo group were withdrawn from the trial because of poor adherence to the trial‐group assignment. Also, one in the propolis group discontinued the trial for a reason unrelated to the study (Figure 1).

### 2.5 Anthropometric assessment

Before and after the intervention period, body weight, fat mass (FM), and fat‐free mass (FFM) were measured using the bioelectrical impedance analysis (BIA) technique (Tania BC‐ 418 machine, Tania Corp., Tokyo, Japan). All participants were asked to be well hydrated and to abstain from exercising and caffeine‐containing products in the 6 hr and 24 hr before the BIA measure, respectively (Kyle et al., 2004). Height was measured using a portable stadiometer (Seca 213, Hamburg, Germany) without shoes in a standing position to the nearest 1 cm. Then, BMI was computed by dividing weight in kilograms by height in meters squared.

### 2.6 Exercise performance assessment

Aerobic endurance was measured using the Cooper 12‐min run test. This test was designed by Dr. Ken Cooper to evaluate the aerobic capacity of the US military (Cooper, 1968). Cooper's test is a valid and reliable method of estimating the maximum oxygen uptake (VO_2_ max) (Bandyopadhyay, 2015). Before and after the intervention period, participants conducted the 12 min of continuous running in the 400‐m running track, and then, covered distance was used to calculate VO_2_ max according to Cooper's equation as follows:

VO_2_ max (ml/kg/min) ₌ (Distance ‐ 504.9) ÷ 44.73.

We also used the running‐based anaerobic sprint test (RAST) to measure anaerobic endurance before and after the intervention period. This test provides a valid and reliable method for estimating anaerobic power and fatigue index (Zagatto et al., 2009). The RAST test comprises six consecutive 35‐m sprints with a rest time of 10 s between sprints. Participants conducted the RAST test, and time spent in each attempt was automatically recorded using an electronic timing system. Then, we computed anaerobic power output along with fatigue index (FI) as the indicator of the drop in power by following equations (Adamczyk, 2011):

- Power output (watts) ₌ (Weight ×Distance ^2^) / Time ^3^
- FI (watts/s) = (Maximum Power – Minimum Power) / Time spent in six sprints

In the familiarization session, all participants were informed about doing sports tests and were motivated to do them with maximal effort. They also were abstained from intense physical activities in the 24 hr before sports tests. All participants had a five‐minute warm‐up period of light aerobic activities prior to performing exercise tests and were verbally encouraged throughout performing exercise tests.

### 2.7 Biochemical assessment

Heparinized blood samples were taken from the cubital vein immediately after Cooper's test. Then, samples were centrifuged at 3,000 rpm for 10 min at 4°C, and supernatants were used to assess inflammation, antioxidants, and oxidative stress. Plasma levels of the interleukin (IL) 6 and IL‐10 were measured based on the biotin double antibody sandwich method using commercial enzyme‐linked immunosorbent assay (ELISA) kits (Diaclone, Besancon, France). Then, the ratio of the IL‐6 and IL‐10 was used to indicate the degree of inflammation status. Plasma levels of total antioxidants capacity (TAC), GSH, and total oxidant status (TOS) were measured based on the enzymatic colorimetric method using commercial kits (ZellBio GmbH, Ulm, Germany). The ratio of the TAC to TOS was used to compute the oxidative stress index (OSI) as follows (Buico et al., 2009): OSI =100 × (TOS/TAC). In addition, malondialdehyde (MDA) concentrations as an indicator of the degree of oxidative damage were assessed by the colorimetric reagent kit (ZellBio GmbH, Ulm, Germany). All tests were read using an ELISA reader (Stat Fax 2,100, Awareness Technology, Inc., USA).

### 2.8 Statistical analysis

Data were analyzed using SPSS software, version 16 (SPSS Inc., Chicago, IL, USA). We estimated that a sample of 27 subjects in each study arm would provide a power of 80% to detect an effect size of 1.4 gr/L in GSH concentrations response to propolis intake (Zhao et al., 2016) and a withdrawal rate of 10% at a significance level of 5% (two‐tailed). Kolmogorov–Smirnov test was used to examine the normal distribution of quantitative data. Within‐group comparisons were done using paired Student's *t* test for normally distributed data and Wilcoxon rank‐sum test for ordinal or non‐normally distributed data. Independent Student's *t* test for normally distributed data, Mann–Whitney U test for ordinal or non‐normally distributed data, and the chi‐square test or Fisher's exact test for nominal data were also used to ascertain between‐group comparisons. We estimated the adjusted effects of propolis using analysis of Covariance (ANCOVA) test with baseline values as covariates. P‐values of less than 0.05 were considered to indicate statistically significant differences.

## 3 RESULTS

The participants had a mean age of 24.21 ± 2.09 years and a BMI of 23.52 ± 1.31 kg/m^2^. The mean age and BMI of participants were 24.21 ± 1.98 years and 23.82 ± 1.06 kg/m^2^ in the propolis group and 24.20 ± 2.24 years and 23.22 ± 1.47 kg/m^2^ in the placebo group. There were no significant differences in age and BMI between the two groups (P‐value >0.05). The mean compliance rate was 96.9% for propolis and 97.1% for placebo. Participants throughout the trial reported no adverse effects.

The adjusted mean changes in the anthropometric characteristics are shown in Table 1. The baseline values of the anthropometric variables were similar in both groups (P‐value >0.05). The mean weight and FM reduced significantly in the placebo group, while no significant changes were observed in the propolis group throughout the trial. However, mean changes in weight and FM did not differ significantly in the placebo group than the propolis group after the adjustment for the baseline values (P‐value >0.05).

**TABLE 1:** Adjusted changes in the anthropometric variables from baseline to the end of the trial

Variables	Group	Before		After	P‐value^a^	Changes^b^	P‐value^b^
Weight; kg	Propolis (*N*:24)		74.42 ± 2.86	74.19 ± 3.24	0.323	−0.244 ± 0.049	0.562
	Placebo (*N*:25)		72.73 ± 3.35	72.29 ± 3.42	0.039	−0.430 ± 0.044	
FM; kg	Propolis (*N*:24)		18.47 ± 4.83	18.44 ± 4.70	0.887	−0.058 ± 0.036	0.294
	Placebo (*N*:25)		20.33 ± 4.97	19.98 ± 4.96	0.011	−0.324 ± 0.034	
FFM; kg	Propolis (*N*:24)		55.85 ± 5.55	55.75 ± 5.94	0.393	−0.251 ± 0.043	0.483
	Placebo (*N*:25)		52.50 ± 6.38	52.31 ± 6.52	0.613	−0.040 ± 0.042	

Note:

Data are presented as mean ±standard deviation.

Abbreviations: FFM, fat‐free mass; FM, fat mass.

^a^ Values were obtained from paired‐sample *t* test;

^b^ Values were obtained from ANCOVA test with baseline values as a covariate.

The adjusted mean changes in athletic performances (aerobic & anaerobic) are shown in Table 2. There were no significant differences in the mean aerobic and anaerobic parameters between the two groups at the baseline examination (P‐value >0.05). At the end of the trial, only mean VO_2_ MAX significantly changed in the propolis group at the end of the trial. However, this change was not significant as compared with the placebo group after the adjustment for baseline values and changes in FFM (P‐value: 0.772). Also, the mean changes in anaerobic Powers and Fatigue Index did not differ significantly from baseline to the end of the trial in both groups (P‐value>0.05).

**TABLE 2:** Adjusted mean changes in the Cooper and RAST tests from baseline to the end of the trial

Variables	Group	Before	After	P‐value^a^	Changes^b^	P‐value^b^
VO_2 MAX_; ml/kg/min	Propolis (*N*:24)	48.04 ± 2.19	49.54 ± 2.38	0.026	1.25 ± 0.109	0.772
	Placebo (*N*:25)	49.12 ± 2.63	49.84 ± 3.05	0.212	0.99 ± 0.107	
Power _MIN_; watts	Propolis (*N*:24)	346.48 ± 75.65	335.01 ± 54.78	0.534	−1.46 ± 3.09	0.118
	Placebo (*N*:25)	323.59 ± 76.79	365.75 ± 86.94	0.055	32.54 ± 2.97	
Power _MEAN_; watts	Propolis (*N*:24)	459.08 ± 48.96	457.68 ± 53.61	0.902	2.41 ± 3.03	0.207
	Placebo (*N*:25)	439.78 ± 50.93	473.03 ± 89.25	0.079	29.59 ± 2.95	
Power _MAX_; watts	Propolis (*N*:24)	654.74 ± 146.43	680.66 ± 138.29	0.482	42.77 ± 6.17	0.734
	Placebo (*N*:25)	619.75 ± 116.01	664.19 ± 152.61	0.265	28.25 ± 5.92	
Fatigue Index; watts/s	Propolis (*N*:24)	8.71 ± 4.13	9.92 ± 3.94	0.297	1.389 ± 0.169	0.292
	Placebo (*N*:25)	8.39 ± 3.34	8.74 ± 4.01	0.753	0.155 ± 0.162	

Abbreviations: FFM, fat‐free mass; FM, fat mass; RAST, Running‐based Anaerobic Sprint Test.

Note: Data are presented as mean ±standard deviation.

^a^ Values were obtained from paired‐sample *t* test;

^b^ Values were obtained from ANCOVA test with baseline values and changes in fat‐free mass as covariates.

The adjusted mean changes in the biochemical parameters measured immediately after Cooper's test are shown in Table 3. The baseline values did not differ significantly between the two groups (P‐value>0.05). The mean IL‐6, IL‐6/IL‐10, MDA, and TOS significantly reduced, while GSH and TAC significantly increased in the propolis group at the end of the trial. But, these variables did not have significant changes in the placebo group. The adjusted mean changes in GSH (Effect Size±Std. Error: 50.39 ± 13.46 µmol/L; P‐value: 0.001), TAC (Effect Size±Std. Error: 259.83 ± 82.7 µmol/L; P‐value: 0.003), IL‐6 (Effect Size±Std. Error: −2.01 ± 0.731 pg/ml; P‐value: 0.011), IL‐6/IL‐10(Effect Size±Std. Error: −0.229 ± 0.101; P‐value: 0.041), MDA (Effect Size±Std. Error: −0.53 ± 0.18 µmol/L; P‐value: 0.011), and TOS (Effect Size±Std. Error: −4.92 ± 1.46 µmol/L; P‐value: 0.001) were significant in the propolis group compared with the control group. Also, significant reductions in OSI (Effect Size: 0.64 ± 0.28; P‐value: 0.032) were observed in the propolis group compared with the control group after the adjustment for baseline values and weight changes.

**TABLE 3:** Adjusted changes in the biochemical assessments from baseline to the end of the trial

Variables	Group	Before	After	P‐value^a^	Changes^b^	P‐value^b^
IL−10; pg/ml	Propolis (*N*:24)	9.95 ± 1.65	10.49 ± 2.01	0.245	0.489 ± 0.085	0.848
	Placebo (*N*:25)	10.15 ± 4.21	10.21 ± 4.19	0.893	0.067 ± 0.082	
IL−6; pg/ml	Propolis (*N*:24)	10.30 ± 3.38	8.85 ± 2.96	0.011	−1.43 ± 0.108	0.011
	Placebo (*N*:25)	9.93 ± 2.92	10.65 ± 3.67	0.247	0.578 ± 0.106	
GSH; µmol/L	Propolis (*N*:24)	229.18 ± 32.06	277.85 ± 62.48	0.001	48.72 ± 2	0.001
	Placebo (*N*:25)	244.87 ± 53.39	243.24 ± 52.55	0.811	−1.70 ± 1.87	
TAC; µmol/L	Propolis (*N*:24)	1,105 ± 357	1,259 ± 447	0.026	164 ± 12	0.003
	Placebo (*N*:25)	1,083 ± 496	1,017 ± 392	0.280	−96 ± 11	
TOS; µmol/L	Propolis (*N*:24)	13.72 ± 4.23	9.13 ± 3.56	0.001	−3.94 ± 0.199	0.001
	Placebo (*N*:25)	11.81 ± 4.85	13.40 ± 6.44	0.200	0.969 ± 0.191	
MDA; µmol/L	Propolis (*N*:24)	3.25 ± 1.22	2.68 ± 0.92	0.005	−0.523 ± 0.029	0.011
	Placebo (*N*:25)	3.07 ± 1.16	3.13 ± 0.87	0.761	0.008 ± 0.028	
IL−6/IL−10	Propolis (*N*:24)	1.05 ± 0.39	0.88 ± 0.41	0.033	−0.174 ± 0.015	0.041
	Placebo (*N*:25)	1.09 ± 0.46	1.14 ± 0.45	0.515	0.051 ± 0.014	
OSI	Propolis (*N*:24)	1.42 ± 0.74	1.00 ± 1.02	0.069	−0.453 ± 0.042	0.032
	Placebo (*N*:25)	1.49 ± 1.27	1.65 ± 1.17	0.494	0.199 ± 0.041	

Note

Abbreviations: GSH, glutathione; IL, interleukin; MDA, malondialdehyde; OSI, oxidative stress index; TAC, total antioxidant capacity; TOS, total oxidant status.

Note: OSI was calculated as follows: 100 × (TOS/TAC). Data are presented as mean ±standard deviation.

^a^ Values were obtained from paired‐sample *t* test;

^b^ Values were obtained from ANCOVA test with baseline values and changes in weight as covariates.

## 4 DISCUSSION

**FIGURE 2:**
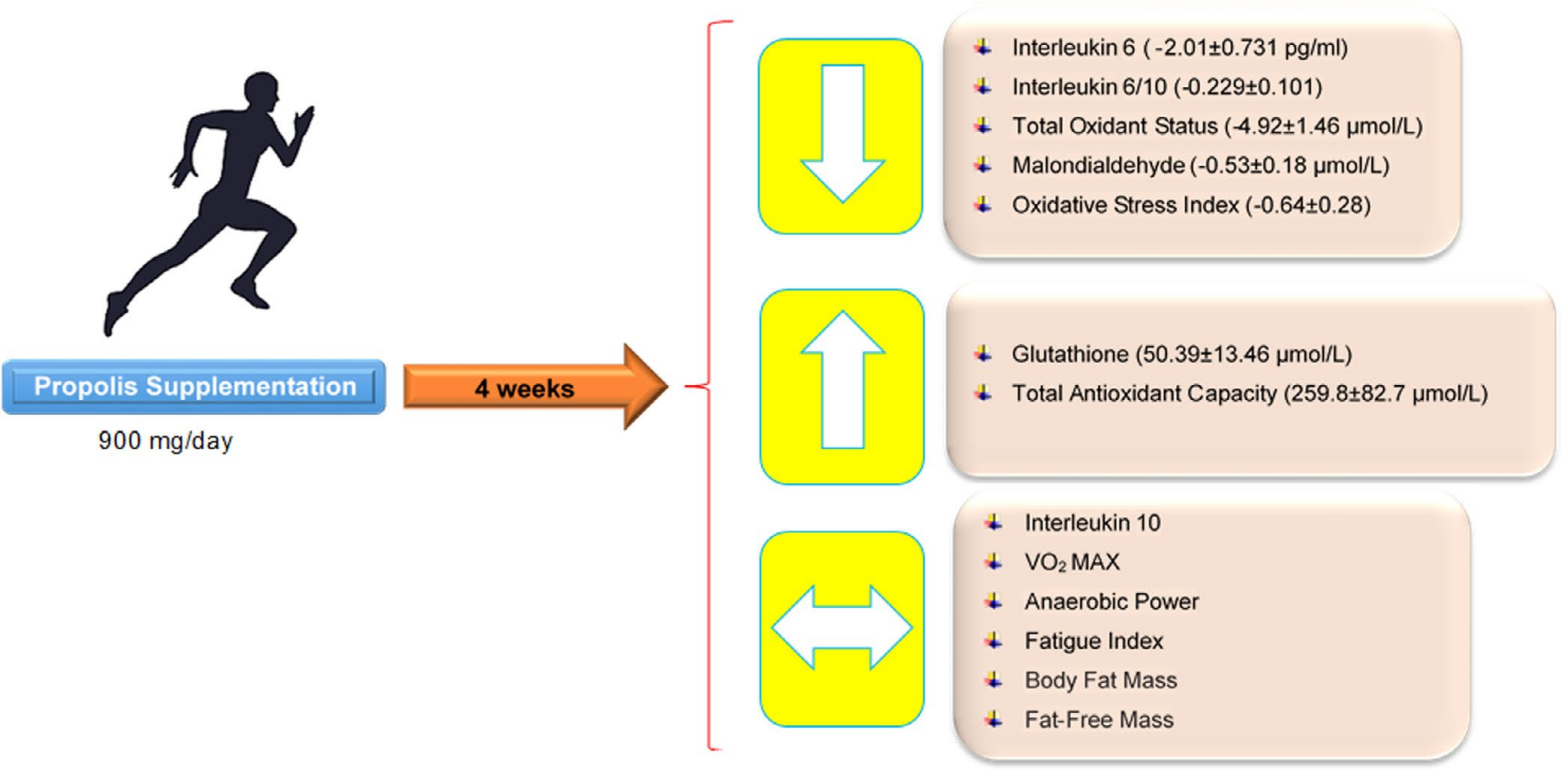
The Effect of Propolis Supplementation on Athletic Performance, Body Composition, Inflammation, and Oxidative Stress following intense exercise. [Down Arrow (Decrease); Up Arrow (Increase); Double‐sided Arrow (Without Change)]

This trial's main finding was that the administration of propolis at a daily dose of 900 mg for four weeks enhanced the antioxidant status and reduced oxidative stress and inflammation following intense exercise. Nevertheless, propolis had no significant effects on body composition and anaerobic and aerobic endurance (Figure 2). To our knowledge, this is the first trial to evaluate the efficacy of propolis supplementation on exercise performance.

Our trial shows that propolis administration has no effects on weight, FFM, and FM in subjects within the normal weight range. In a 4‐month randomized clinical trial, Soleimani et al. found that supplementation with 500 mg/day of propolis had no significant effect on weight, FFM, and FM among patients with nonalcoholic fatty liver disease (Soleimani, Rezaie, et al., 2021). Likewise, Mujica et al. reported that the administration of 30 drops/day of propolis solution (Beepolis®) for three months had no effects on weight and waist circumference among patients with cardiometabolic risk factors (Mujica et al., 2017). Also, Zakerkish et al. showed that the daily intake of 1,000 mg/day of propolis for three months had no effects on weight in diabetic subjects (Zakerkish et al., 2019). Conversely, Samadi et al. reported that the administration of 900 mg/day of propolis in diabetic subjects over a period of 3 months reduced weight and BMI while did not affect waist circumference (Samadi et al., 2017). This controversy can be related to confounders' effect, such as changes in energy intake and physical activity throughout the study of Samadi et al.

Our trial shows that propolis supplementation improves TAC and GSH and reduces TOS, MDA, and OSI following intense exercise. These findings on the improvement of the antioxidant defense system's capacity are in line with the observations of previous clinical trials. In an 18‐week randomized clinical trial, Zhao et al. reported that among diabetic subjects, propolis supplementation at a daily dose of 900 mg/day elevated serum levels of GSH and total polyphenols (Zhao et al., 2016). In the study of Mujica et al., the administration of propolis significantly increased the levels of GSH in red blood cells (RBCs) and decreased the plasma MDA concentrations after 3 months (Mujica et al., 2017). Likewise, Hesami et al. showed that 1,500 mg/day of propolis administration significantly increased catalase activity and reduced oxidized low‐density lipoprotein (LDL) concentrations in diabetic subjects (Hesami et al., 2019). Propolis contains high amounts of phenolic compounds which directly react with and quench free radicals. In addition, propolis and its derivatives have been shown to activate the nuclear factor erythroid‐2‐related factor 2 (Nrf2) pathway, which mainly regulates the expression of a large battery of genes related to endogenous antioxidants (Jin et al., 2015). In this line, propolis has been shown in vivo to increase the levels of heme oxygenase‐1 (HO‐1), glutamate‐cysteine ligase (GCL: the rate‐limiting enzyme for the biosynthesis of GSH), and thioredoxin reductase 1 (TrxR1) (Zhang et al., 2015). The activation of the Nrf2 pathway not only up‐regulates the GCL mRNA expression but also facilitates the cellular uptake of cystine as the rate‐limiting precursor for the synthesis of GSH via enhancing the cystine/glutamate amino acid antiporter (Xc‐system) (Correa et al., 2011).

Our trial shows that propolis supplementation reduces the IL‐6 concentrations and IL‐6/IL‐10 ratio in plasma following intense exercise. Intense physical activities stimulate inflammatory cytokines, which attenuate muscle force production, immune response, and recovery times (Nemet et al., 2002). There are some inconsistencies regarding the effect of propolis on inflammatory cytokines in previous clinical trials. Zakerkish et al.'s study showed that propolis supplementation reduced TNF‐α while had no effect on IL‐6 and IL‐1β among diabetic subjects (Zakerkish et al., 2019). The study of Zhao et al. also reported that propolis reduced TNF‐α concentrations, whereas increased IL‐1β and IL‐6 levels among diabetic subjects (Zhao et al., 2016). Fukuda et al. found that propolis supplementation (226.8 mg/day for 8 weeks) had no effect on TNF‐α and IL‐6 concentrations among diabetic subjects (Fukuda et al., 2015). Khayyal et al. found that propolis supplementation (260 mg/day for 8 weeks) reduced TNF‐α, intercellular adhesion molecule‐1 (ICAM‐1), IL‐6, and IL‐8 and increased IL‐10 in asthmatic patients (Khayyal et al., 2003). Afsharpour et al. reported that propolis supplementation (1,500 mg/day for 8 weeks) reduced TNF‐α among diabetic subjects (Afsharpour et al., 2017). Taken together, a recent meta‐analysis revealed a significant reduction in IL‐6, TNF‐α, and high‐sensitivity C‐reactive protein (hs‐CRP) concentrations by 17.96 pg/ml, 34.08 pg/ml, and 1.16 pg/ml, respectively, following propolis supplementation. Nonetheless, no significant reduction was reported in IL‐1β concentrations with propolis consumption (Shang et al., 2020). Recent scientific evidence (in vitro and in vivo) shows that propolis and its bioactive compounds attenuate inflammatory cytokines synthesis and leukocyte recruitment into the inflammatory site through various mechanisms of action such as blocking the activation of NF‐κB, extracellular signal‐regulated kinase (ERK), and Jun N‐terminal Kinase (JNK) signaling pathways, inhibiting the release of CXCL1/KC and CXCL2/MIP‐2 chemokines, and suppressing the expression of ICAM‐1, vascular cell adhesion molecule 1 (VCAM‐1), and E‐selectin (Franchin et al., 2018). Nuclear factor‐kappa B (NF‐κB) is a critical transcription factor for the activation of inflammatory gene expression in response to various stimuli such as ROS/RNS. Caffeic acid phenyl ester which is identified in propolis has been shown to reduce exercise‐induced skeletal muscle injury by inhibiting the activation of NF‐κB as well as the generation of ROS (Shen et al., 2013). Collectively, propolis may be a promising candidate to treat acute and chronic inflammatory diseases (Franchin et al., 2018; Soleimani, Miryan, et al., 2021).

Our results failed to show a significant improvement in aerobic and anaerobic endurance. Scientific evidence on the effect of antioxidants on exercise performance is limited and conflicting. In a 1‐week randomized trial, Davis et al. found that the daily administration of 1,000 mg of quercetin, which is identified in propolis, increased VO_2_ max (3.9%) and bike‐ride times to fatigue (13.2%) among untrained subjects (Davis et al., 2010). In a 3‐week randomized trial, Jourkesh et al. reported that a daily intake of 400 mg of vitamin E along with 1,000 mg of vitamin C improved VO_2_ max. However, it had no significant effect on anaerobic power among male students (Jourkesh et al., 2007). Conversely, the administration of 1,000 mg/day of vitamin C for 4 weeks had no significant effect on VO_2_ max among recreationally active men (Roberts et al., 2011). Likewise, in another clinical trial, Paulsen et al. reported that the daily intake of 1,000 mg of vitamin C and 235 mg of vitamin E for 11 weeks did not affect VO_2_ max on healthy subjects (Paulsen et al., 2014). Taken together, it seems that long‐term antioxidant supplementation may attenuate its favorable effects on exercise performance. In this line, a body of evidence suggests that high levels of antioxidants in skeletal muscles can attenuate exercise adaptations and mitochondrial biogenesis by suppressing redox signaling pathways related to exercise‐induced ROS production (Cobley et al., 2015; Strobel et al., 2011). Consistent with this hypothesis, it has been shown that ROS can modulate the force production in unfatigued skeletal muscles (Powers & Jackson, 2008).

The lack of access to an ergo‐spirometer for direct evaluation of VO_2_ max via a breath‐by‐breath gas analyzing system was a limitation of this study. However, we used the Cooper test to evaluate the aerobic capacity of the military (Cooper, 1968). This test is a valid and reliable method for the estimation of VO_2_ max in the field (Bandyopadhyay, 2015). Another limitation of this study is that it was conducted only among men and the results may not be generalizable to women. This trial's strengths were the triple‐blind design, high compliance rates, matched control, the similarity in terms of living place, physical activities, meals, and sleep duration among all participants, minimal drop‐out rates, and adjustment for potential confounding factors.

In conclusion, our findings demonstrate that supplementation with propolis might have beneficial effects on oxidative stress and inflammation status following intense physical activities while not affecting athletic performance in healthy active subjects. Further studies are needed to ascertain the effect of propolis on exercise performance in trained and nontrained subjects.

## CONFLICT OF INTEREST

The authors have declared no conflict of interest.

## ETHICS APPROVAL

The trial protocol was approved by the research ethics committee by the ethics committee at the AJA University of Medical Sciences (IR.AJAUMS.REC.1399.107). All patients provided written consent for participation in this study.

## Data Availability

The data that support the findings of this study are available on request from the corresponding author. The data are not publicly available due to privacy or ethical restrictions.
